# Supplementary material for: UTMOST, a single and cross-tissue TWAS (Transcriptome Wide Association Study), reveals new ASD (Autism Spectrum Disorder) associated genes
Source: Transl Psychiatry. 2021 Apr 30;11:256. doi: 10.1038/s41398-021-01378-8 (PMC8087708; doi:10.1038/s41398-021-01378-8)

Supplementary Figure 1a. Regional plots using ASD metaanalysis summary statistics for the UTMOST associated genes in brain tissues (*NKX2-2, PTPRE*).

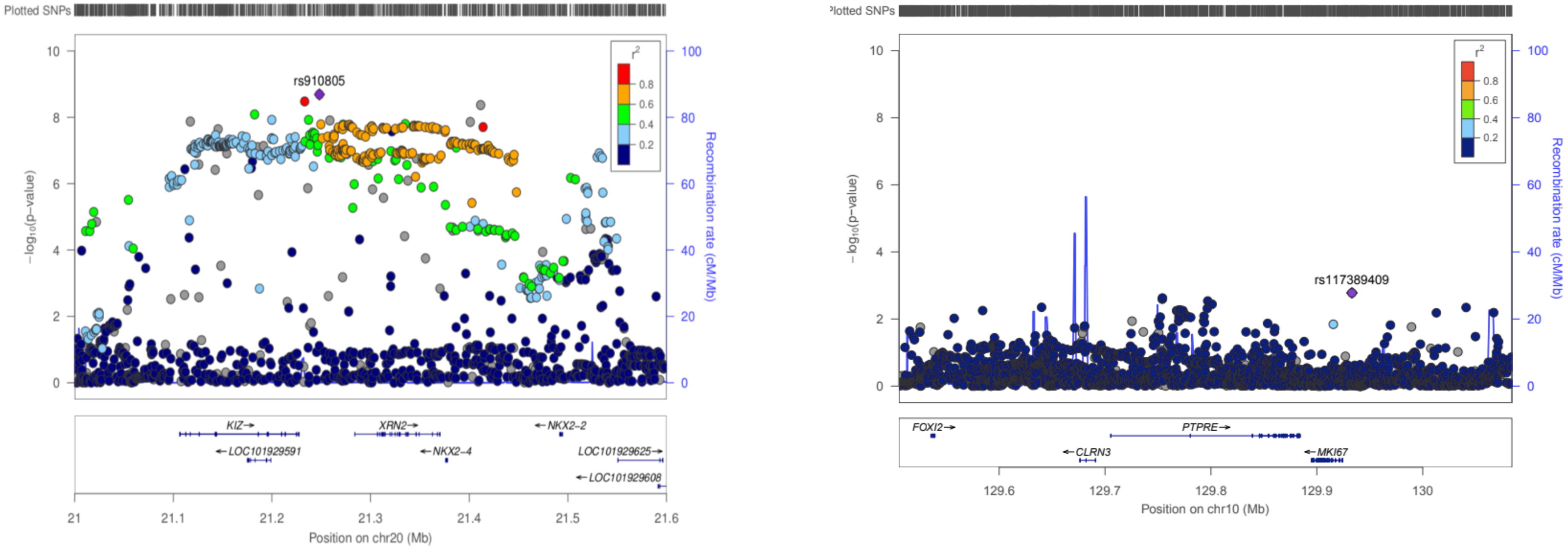

Supplementary Figure 1b. Regional plots using ASD metaanalysis summary statistics for the UTMOST associated genes in brain tissues (*CIPC*, *PINX1*).

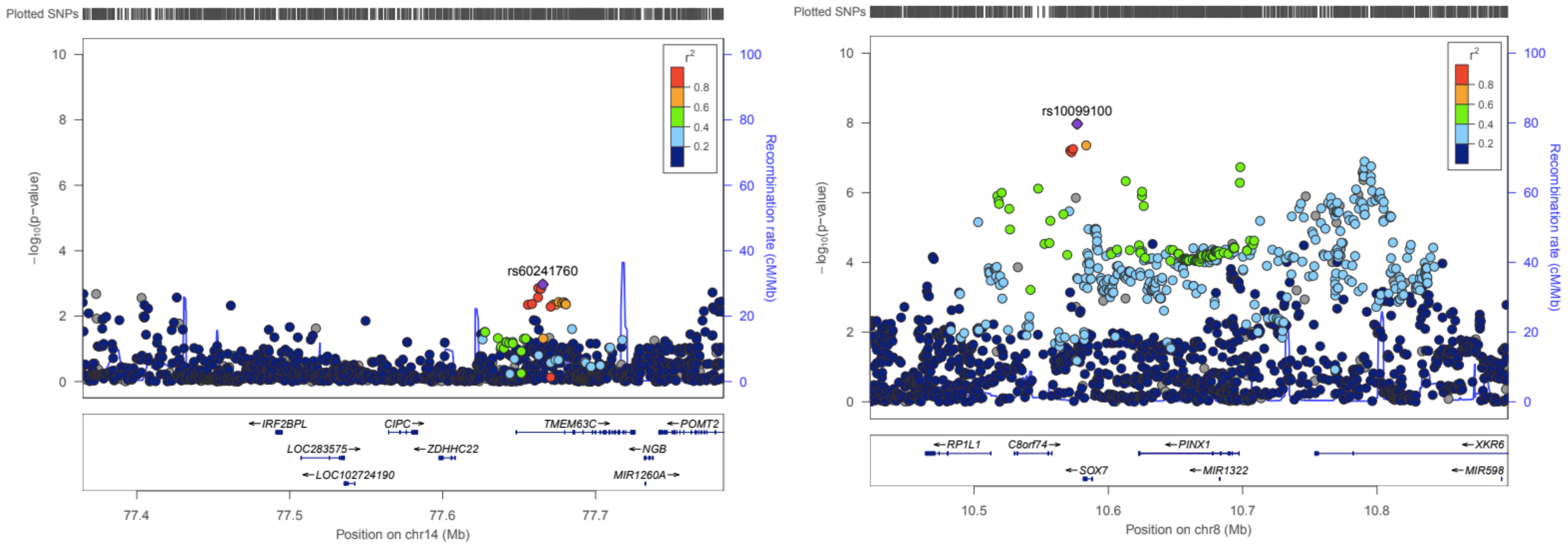

Supplementary Figure 2a. Regional plots using ASD metaanalysis summary statistics for the UTMOST associated genes in gastrointestinal tissues (*MANBA, ERI1*). *NKX2-2* has shown association in this analysis but the regional plot is included in the previous Supplementary Figure 1.

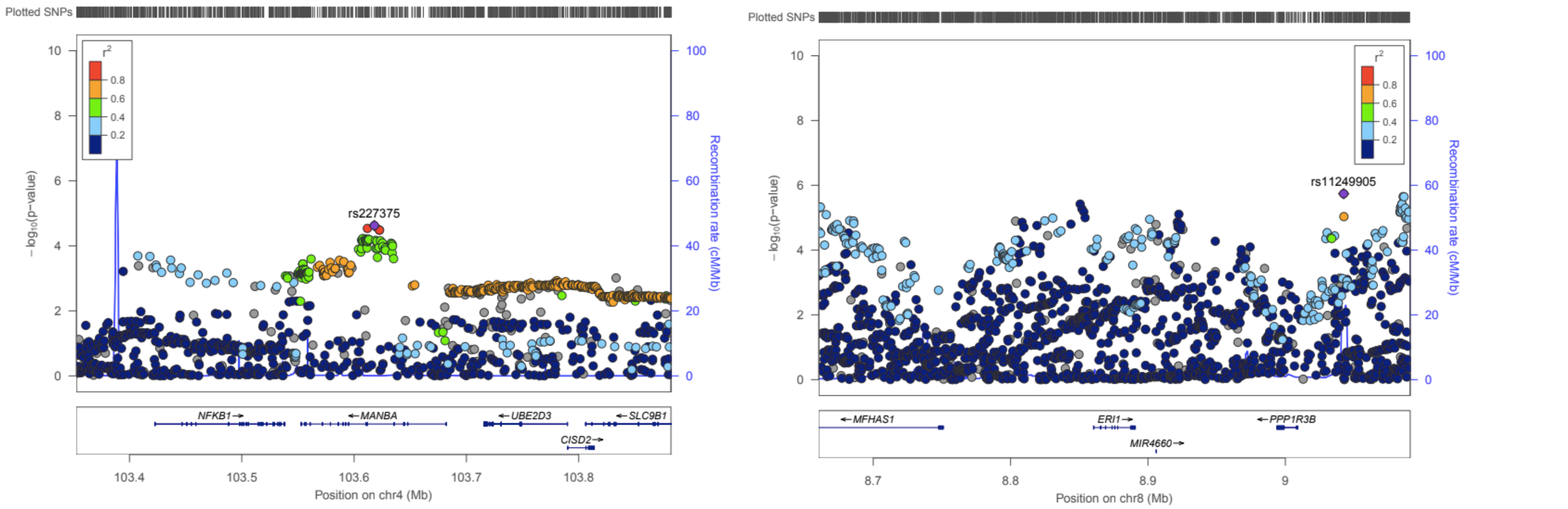

Supplementary Figure 2b. Regional plots using ASD metaanalysis summary statistics for the UTMOST associated gene in gastrointestinal tissues *MITF* and cross-tissue analysis *BLK* . *NKX2-2* is also associated in these analysis but the regional plot is included in the Supplementary Figure 1.

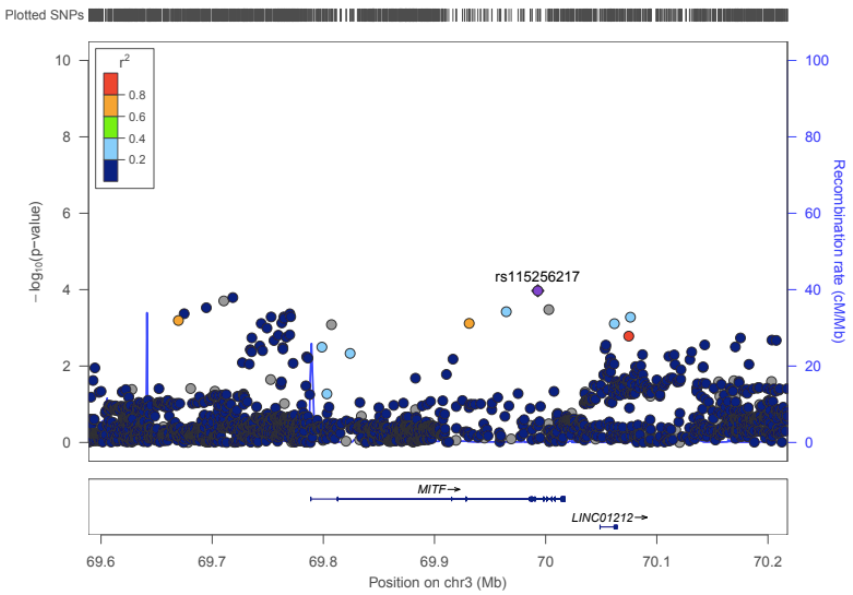

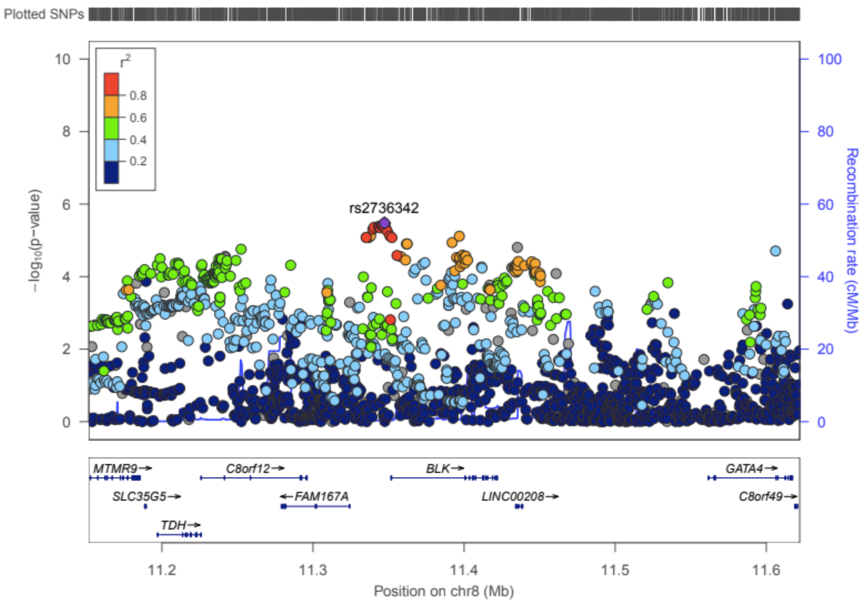

Supplementary Figure 3. UCSC screenshot showing the location of SE_06106 superenhancer on CIPC gene (hippocampus).

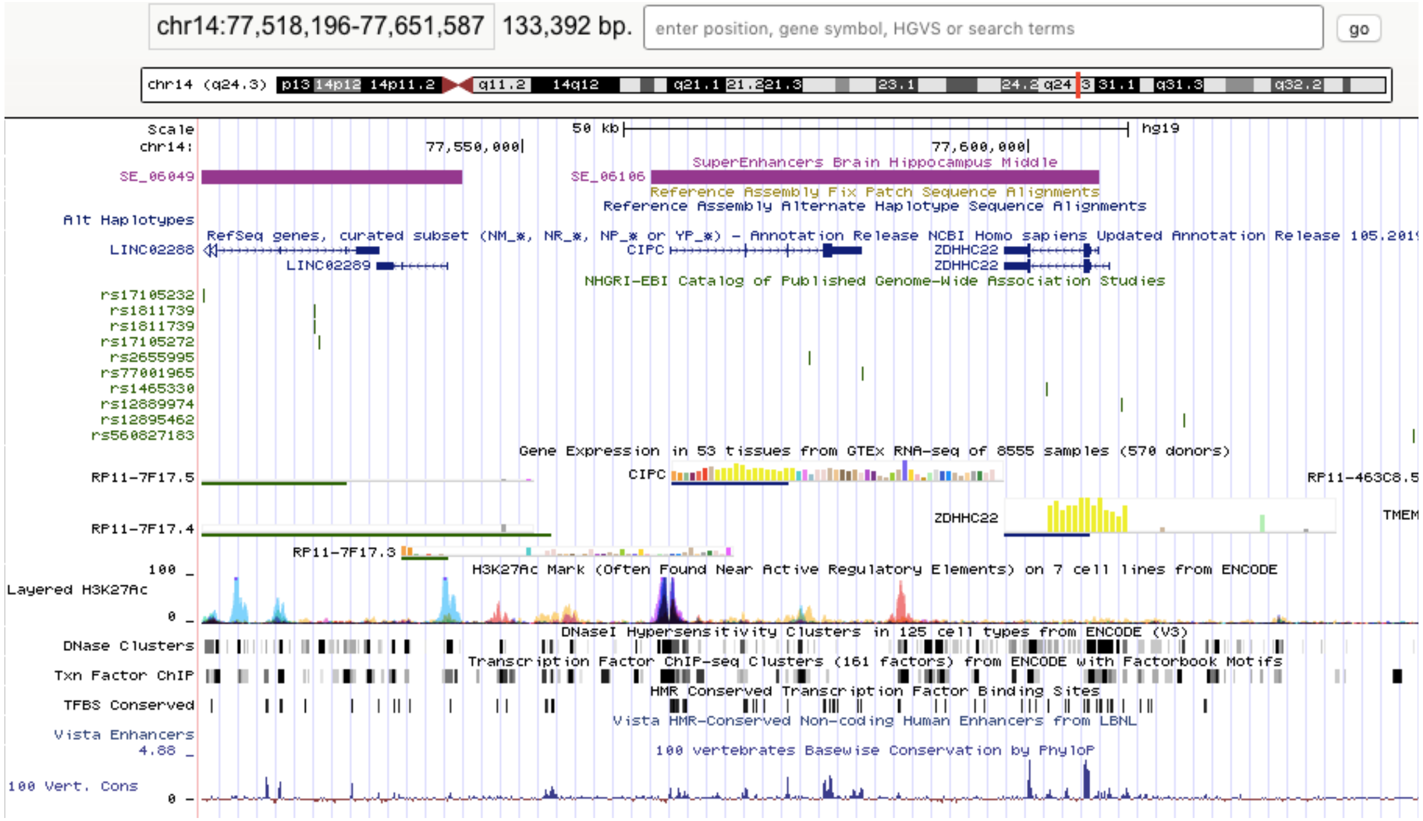

Supplement: Supplementary file 1 — Supplementary Figures [file 41398_2021_1378_MOESM1_ESM.pdf]
